# Diagnostic efficiency of intravoxel incoherent motion-based virtual magnetic resonance elastography in pulmonary neoplasms

**DOI:** 10.1186/s40644-024-00728-1

**Published:** 2024-07-06

**Authors:** Shuo Zhang, Yonghao Du, Ting Liang, Xuyin Zhang, Yinxia Guo, Jian Yang, Xianjun Li, Gang Niu

**Affiliations:** Department of Radiology, the First Affiliated Hospital of Xi’an Jiaotong University, 277 Yanta West Road, Yanta District, Xi’an, 710061 P.R. China

**Keywords:** Intravoxel incoherent motion, Virtual magnetic resonance elastography, Stiffness, Shifted apparent diffusion coefficient, Pulmonary neoplasm

## Abstract

**Background:**

The aim of the study were as below. (1) To investigate the feasibility of intravoxel incoherent motion (IVIM)-based virtual magnetic resonance elastography (vMRE) to provide quantitative estimates of tissue stiffness in pulmonary neoplasms. (2) To verify the diagnostic performance of shifted apparent diffusion coefficient (sADC) and reconstructed virtual stiffness values in distinguishing neoplasm nature.

**Methods:**

This study enrolled 59 patients (37 males, 22 females) with one pulmonary neoplasm who underwent computed tomography-guided percutaneous transthoracic needle biopsy (PTNB) with pathological diagnosis (26 adenocarcinoma, 10 squamous cell carcinoma, 3 small cell carcinoma, 4 tuberculosis and 16 non-specific benign; mean age, 60.81 ± 9.80 years). IVIM was performed on a 3 T magnetic resonance imaging scanner before biopsy. sADC and virtual shear stiffness maps reflecting lesion stiffness were reconstructed. sADC and virtual stiffness values of neoplasm were extracted, and the diagnostic performance of vMRE in distinguishing benign and malignant and detailed pathological type were explored.

**Results:**

Compared to benign neoplasms, malignant ones had a significantly lower sADC and a higher virtual stiffness value (*P* < 0.001). Subsequent subtype analyses showed that the sADC values of adenocarcinoma and squamous cell carcinoma groups were significantly lower than non-specific benign group (*P* = 0.013 and 0.001, respectively). Additionally, virtual stiffness values of the adenocarcinoma and squamous cell carcinoma subtypes were significantly higher than non-specific benign group (*P* = 0.008 and 0.001, respectively). However, no significant correlation was found among other subtype groups.

**Conclusions:**

Non-invasive vMRE demonstrated diagnostic efficiency in differentiating the nature of pulmonary neoplasm. vMRE is promising as a new method for clinical diagnosis.

**Supplementary Information:**

The online version contains supplementary material available at 10.1186/s40644-024-00728-1.

## Background

Lung cancer is the most common malignancy and the leading cause of cancer-related death worldwide [1, 2]. Accurate diagnosis of benign and malignant pulmonary neoplasm and identification of pathological type are crucial for clinical decision-making. Biopsy and radiological techniques with contrast administration are mostly necessary for establishing diagnosis, but both have contraindications and complications. Moreover, the diagnostic performance of conventional radiological methods needs to be improved. Due to the limitations of these existing tools, exploring non-invasive techniques with the potential of replacing invasive histopathologic analysis of pulmonary neoplasm remains urgent.

Stiffness is an intrinsic material property of the tissue. Increased tissue stiffness is the most tangible and best-recognized mechanical abnormality in tumors [3]. Magnetic resonance elastography (MRE) was developed to probe the mechanical properties of tissue and allow an estimation of the true viscoelasticity of various tissues and organs. However, due to the poor portability and limited availability of the appropriate magnetic resonance (MR) equipment, the clinical application of MRE has large limitations [4].

Intravoxel Incoherent Motion (IVIM)-based virtual MRE (vMRE) is a novel method with potential application for the non-invasive assessment of tissue stiffness and without the limitation of specialized hardware and expertise of traditional MRE [5]. This method was first proposed by Le Bihan et al. in the evaluation of liver fibrosis, and it was subsequently verified in a small (*n* = 15) [5] and a larger patient cohort (*n* = 74) [6]. These previous studies reveal a strong correlation between tissue water diffusivity and tissue elasticity in the liver. In short, diffusion MR imaging, through a calibration of shifted apparent diffusion coefficient (sADC) values with standard MRE, can be converted quantitatively into shear modulus to provide tissue stiffness information, requiring only two b values [5].

As a non-invasive method that relies on widely available magnetic resonance imaging (MRI) sequences with high image resolution and short acquisition time, the application of vMRE has been extended to other diseases of the liver (e.g., hepatocellular carcinoma, liver metastases [7], non-alcoholic fatty liver disease [8]) and brain (e.g., pituitary adenoma [9] and meningiomas [10]). For other organs and diseases, tissue elastic properties and tissue microstructure should be tightly related, similar to liver tissue. Therefore, we hypothesized that vMRE could provide information for judging pulmonary neoplasms. This vMRE application could make up for the shortcomings of conventional radiological methods by introducing elastic parameters to improve diagnostic efficiency. The aim of this study was to explore the capability of vMRE in the characterization of pulmonary neoplasms through quantitative sADC and virtual stiffness values. To our knowledge, the method has not previously been used in pulmonary neoplasm evaluation.

## Methods

### Study participants

Consecutive pulmonary neoplasm patients who underwent a computed tomography (CT)-guided percutaneous lung biopsy between April 2018 and March 2020 were enrolled in our study. The inclusion criteria were as follows: (1) CT-confirmed primary pulmonary neoplasm; (2) lesion diameter ≥ 3.0 cm; (3) each neoplasm had an exact pathological diagnosis; (4) patient free from any comorbidities or previous cancers. The exclusion criteria were as follows: (1) the boundary between the lesion and adjacent structures was unclear; (2) the images were blurred with obvious artifacts; (3) patient had already received antitumor therapy prior to IVIM examination; (4) pulmonary metastasis (primary tumors outside the chest).

### MR imaging protocol

MRI was performed using a 3 T Signa HDXT (General Electric, Milwaukee, WI, USA) with an eight-channel body phased-array coil for signal reception. The scanning was stopped immediately once the participants felt uncomfortable. IVIM images were collected with the following b values: 0, 10, 15, 20, 25, 50, 80, 150, 300, 500 and 800 s/mm^2^. All MRI sequences and parameters are listed in Table 1.

**Table 1:** 3.0-T MR imaging sequences and parameters

Parameter	T2-2D FSE	T1-2D FIESTA	IVIM
Respiration pattern	Breath-hold	Breath-hold	Breath-hold
Plane	Axial	Axial	Axial
Repetition time (ms)	8000	3.2	9000
Echo time (ms)	95.1	1.1	65.4
Matrix	512 × 512	512 × 512	512 × 512
Section thickness/slice spacing (mm)	5/0	5/0	4/0
Field of view (mm)	400	350	400

Note FSE: fast spin echo; FIESTA: fast imaging employing steady-state acquisition

### vMRE stiffness quantification

After scanning, sADC and virtual stiffness values were calculated by two key b values (low key b value, LKb, and high key b value, HKb) to estimate the shear modulus of pulmonary neoplasm. The quantification is summarized below (Eqs. 1 and 2) and described in detail by Le Bihan et al. [5].

1 $$\rm sADC \,\left(s/mm^2\right)=ln \,\left(\text{S}_\text {LKb}/\text{S}_\text{HKb}\right)/\left(\text{HKb} - \text{LKb}\right)$$

2 $$\rm virtual \,stiffness \left(kPa\right) = \alpha \cdot\,ln \,\left(\text{S}_\text{low}/\text{S}_\text{high}\right) + \beta$$

S_LKb_ and S_HKb_ represent intensities of the signals acquired at the low and high key b values, which were S_low_ (b value = 150 s/mm^2^) and S_high_ (b value = 800 s/mm^2^) in our study, respectively. The scaling (α) and the shift (β) factors were separately set to -9.8 and 14, respectively, according to the previous calibration studies.

### Image analysis

Two radiologists, each with more than five years of experience in diagnosing chest images, drew the regions of interest (ROI) independently on sADC and shear stiffness maps. The ROIs were placed on three contiguous axial slices, in which the tumors had maximum diameters, and to avoid necrotic, bleeding, or inhomogeneous areas. The average value was recorded from these ROIs. Both radiologists were blinded to the patients’ clinical history and histopathological diagnosis.

The quantification of sADC and virtual stiffness were performed off-line using MATLAB R2016a (Mathworks, Natick, Massachusetts, USA).

### Statistical analysis

After assessing the normal distribution of sADC and virtual stiffness values with the Kolmogorov-Smirnov test, Mann-Whitney U test was used to evaluate differences between benign and malignant pulmonary neoplasm groups. Categorical variables are shown as the number and were compared using the chi-squared test. One-way analysis of variance (ANOVA) was performed to calculate the differential values of each pathological pattern in the malignant groups. The receiver operating characteristic (ROC) curve was used to assess the discrimination capability of the pulmonary neoplasm property. The intra- and inter-observer agreement was determined using intraclass correlation coefficients (ICC) with 95% confidence intervals, where the two-sided random ICC model was used to determine the consistency of the agreement. Statistical analyses were all performed using SPSS software (version 18.0, IBM, Chicago, IL). Statistical significance was accepted as a two-sided *P* value < 0.05.

## Results

### Characteristics of the study participants

**Fig. 1:**
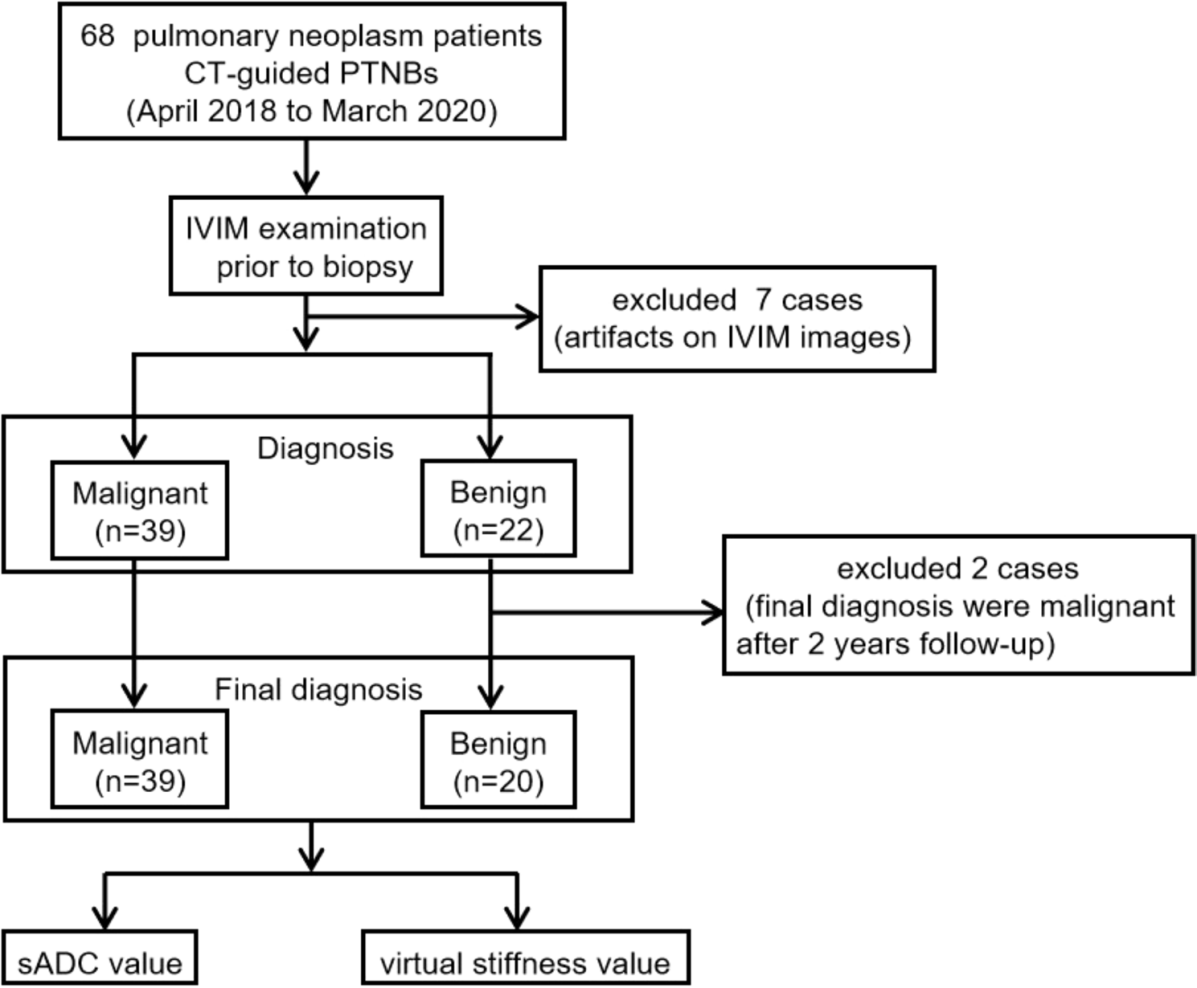
Flow chart of the enrollment process

We recruited 68 individuals for our study. Seven patients were excluded because of artifacts on IVIM images (mainly from respiratory or heart motion). After 2 years follow-up, two non-specific benign cases were excluded because the final diagnosis were malignant. We finally enrolled 59 participants (36 males, 23 females; mean age at diagnosis: 60.81 ± 9.80 years), each with one pulmonary neoplasm for further assessment. The enrollment procedure is presented in Fig. 1. These patients were divided into two groups based on their final histopathological diagnosis. The malignant group (39/59) included patients with adenocarcinoma, squamous cell carcinoma and small-cell carcinoma, whereas the benign group (20/59) included patients with tuberculosis and non-specific benign. Table 2 lists the clinical characteristics of the enrolled patients.

**Table 2:** Clinical characteristics of patients with pulmonary neoplasm

Characteristics	Malignant *n* = 39	Benign *n* = 20	*P*
Age (years)	61.03 ± 10.23	59.94 ± 9.41	0.820
Male/Female	24/15	13/7	0.796
Lesion diameter (cm)	4.93 ± 1.88	4.12 ± 1.11	0.010^*^
Location			0.073
Upper/Middle lobe	27	9	
Lower lobe	12	11	

Note *P value < 0.05 indicates statistical significance. Date are shown as Mean ± SD. Continuous variables were compared using the Mann-Whitney U test. Categorical variables are shown as the number and were compared using the chi-squared test

### Intra- and inter-observer reproducibility agreement

The intra- and inter-observer reproducibility for measurement of sADC and virtual stiffness values are presented in Table 3. Good intra- and inter-observer reproducibility were obtained, with ICC values in the range of 0.864–0.956.

**Table 3:** Intra- and inter-observer reproducibility in Assessment of sADC and virtual stiffness

	ICC (95% CI)	
	Intra-observer	Inter-observer
sADC(s/mm^2^)	0.956 (0.927–0.974)	0.864 (0.782–0.917)
virtual stiffness (kPa)	0.941 (0.903–0.964)	0.887 (0.826–0.928)

Note ICC, intra-class correlation coefficient. 95% CI, 95% confidence interva

### sADC and virtual stiffness values were significantly different in malignant and benign pulmonary neoplasms

**Fig. 2:**
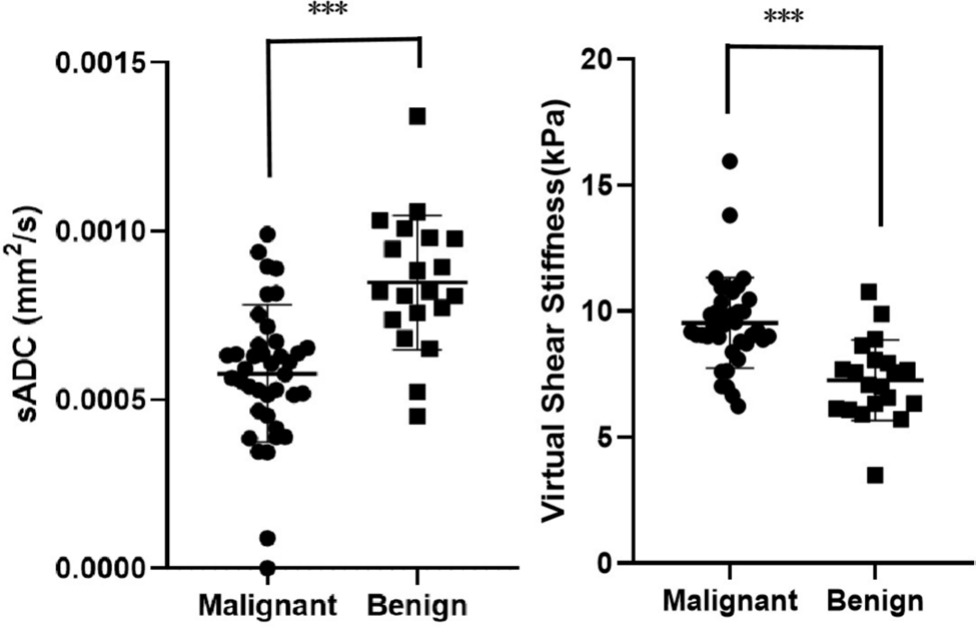
Comparison of sADC and virtual stiffness values between benign and malignant groups. sADC value in malignant group was significantly lower than benign group (*P* < 0.001), while virtual stiffness value in malignant group was significantly higher than benign group (*P* < 0.001)

**Fig. 3:**
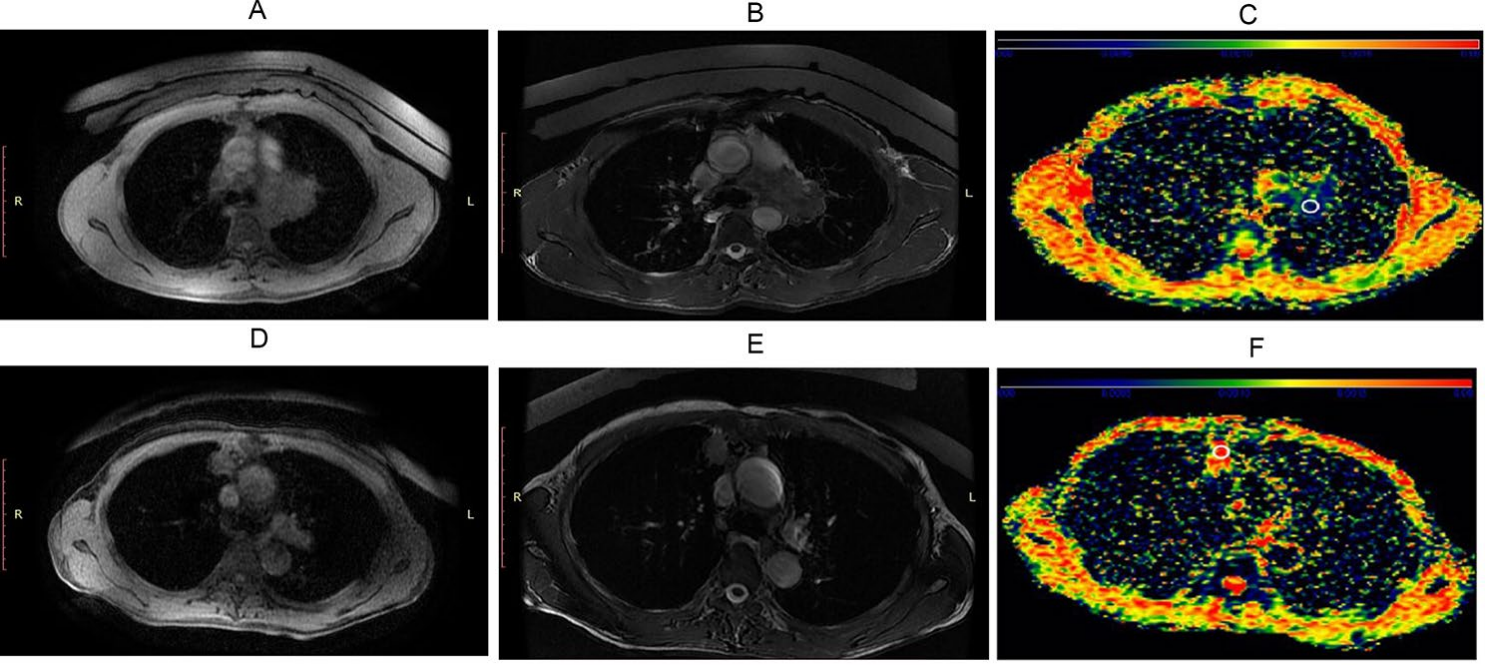
T1-2D FIESTA and T2-2D FSE images together with sADC maps in benign and malignant neoplasms. The adenocarcinoma in the upper lobe of the left lung presented signs of lower sADC and higher virtual stiffness values in sADC map (A-C). The inflammatory nodule in the upper lobe of the right lung displayed signs of higher sADC and lower virtual stiffness values in sADC map (D-F). Regions of interests (ROIs) on C and F (white circle) were set to measure the sADC value of the two cases. FIESTA: fast imaging employing steady-state acquisition; FSE: fast spin echo

**Fig. 4:**
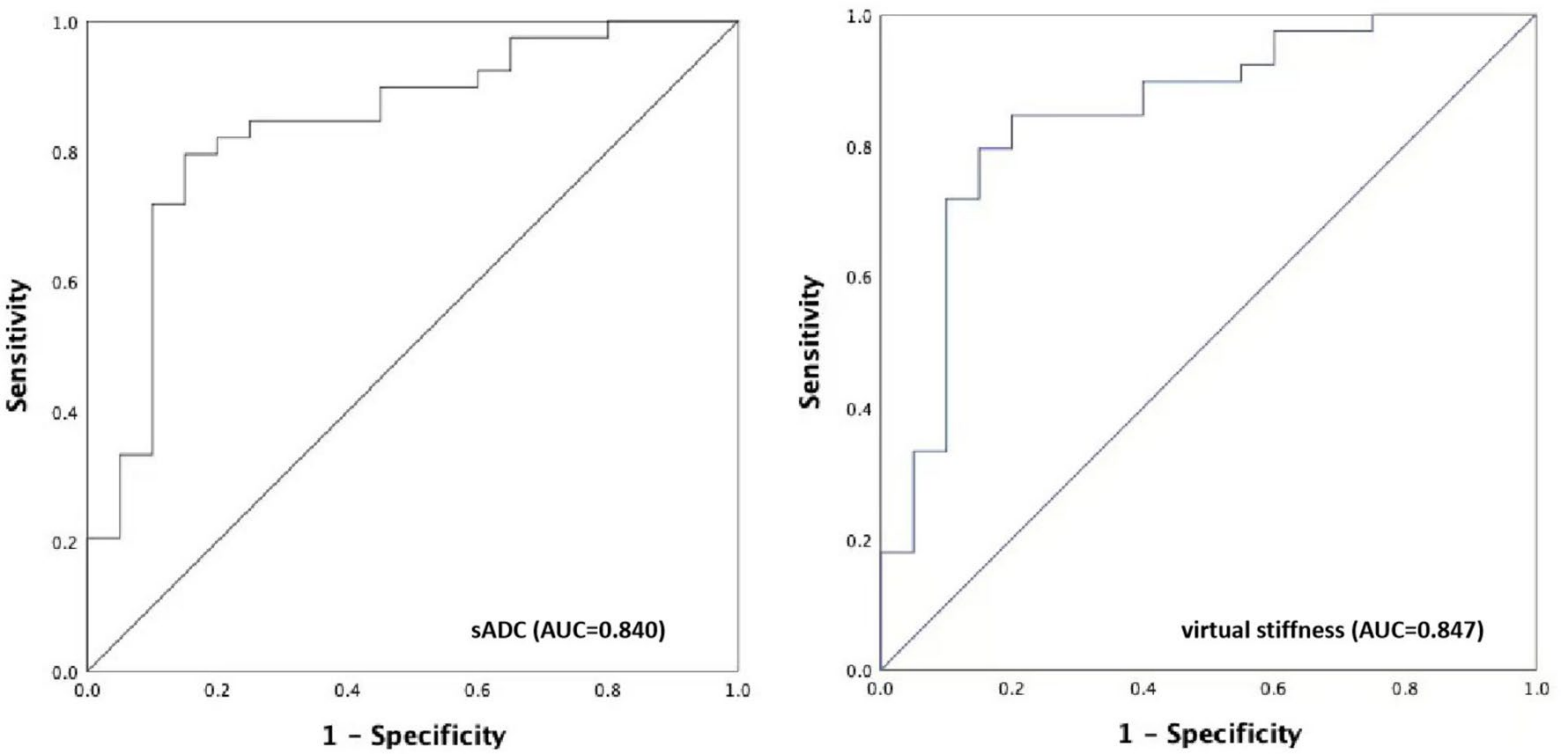
Receiver operating characteristic (ROC) curves of sADC and virtual stiffness. The areas under the ROC curves of the sADC and virtual stiffness were 0.840 and 0.847, respectively. The sensitivity and specificity of sADC were 79.5% and 85.0%, and those of virtual stiffness were 84.6% and 80.0%, respectively

After scanning, sADC and shear stiffness maps were obtained. Next, we compared sADC and virtual stiffness values of each pulmonary neoplasm to determine whether there was any difference between malignant and benign groups. As shown in Table 4; Fig. 2, the mean value of sADC in the malignant group was significantly lower than that in the benign group (5.78 × 10^− 4^ ± 2.03 × 10^− 4^ s/mm^2^ versus 8.47 × 10^− 4^ ± 1.99 × 10^− 4^ s/mm^2^, *P* < 0.001). As for virtual stiffness, a higher value was observed in the malignant group than in the benign group (9.53 ± 1.77 kPa versus 7.27 ± 1.60 kPa, *P* < 0.001). Examples of T1-weighted (T1W) fast imaging employing steady-state acquisition (FIESTA) axial images and fat saturated T2-weighted (T2W) fast spin echo (FSE) axial images together with sADC maps in benign and malignant neoplasms are presented in Fig. 3. An ROC curve was drawn to assess the discrimination capability of pulmonary neoplasm property. The areas under the curve of sADC and virtual stiffness values for distinguishing pulmonary neoplasm nature were 0.840 and 0.847, respectively. The sensitivity and specificity in differentiating malignant from benign neoplasms were 79.5% and 85.0% for sADC versus 84.6% and 80.0% for virtual stiffness values (Fig. 4). The cut-off value of sADC was 6.78 × 10^− 4^ s/mm^2^, while that for the virtual stiffness value was 8.08 kPa. These results indicate that the sADC and virtual stiffness values possess the potential to predict pulmonary neoplasm properties.

**Table 4:** sADC and virtual stiffness of Benign and Malignant neoplasms

	sADC (×10^− 4^, b = 200,800 s/mm^2^) Mean ± SD (95% C.I.)	virtual stiffness (kPa) Mean ± SD (95% C.I.)
Malignant(*n* = 39)	5.78 ± 2.03 (5.12–6.43)	9.53 ± 1.77 (8.96–10.11)
Benign (*n* = 20)	8.47 ± 1.99 (7.54–9.40)	7.27 ± 1.60 (6.52–8.01)
*P* value	<0.001***	< 0.001***

Note *P value < 0.05 indicates statistical significance; ***P < 0.001

### Subtype analyses of sADC and virtual stiffness values

**Fig. 5:**
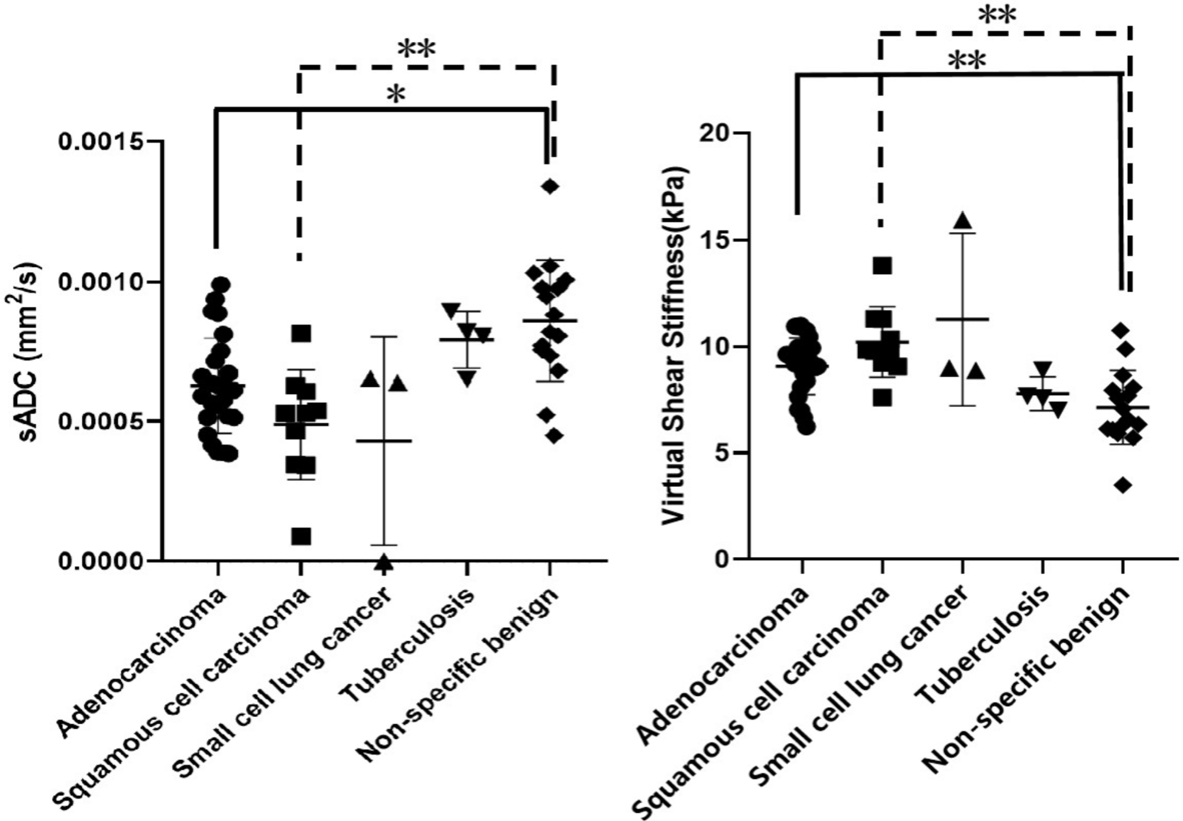
Comparison of sADC and virtual stiffness values for detailed pathological type. sADC value of adenocarcinoma and squamous cell carcinoma groups was lower than that of non-specific benign group (*P* = 0.013 and 0.001, respectively). Virtual stiffness value of the two malignant subtypes is higher than that of non-specific benign group (*P* = 0.008 and 0.001, respectively)

We subsequently compared sADC and virtual stiffness values among each pathological pattern. The data showed that the sADC values of the adenocarcinoma and squamous cell carcinoma groups were lower than the non-specific benign group, while virtual stiffness values were higher in the two subtypes of malignant pulmonary neoplasm. As shown in Table 5; Fig. 5, we found significantly higher sADC values in the non-specific benign group compared to the adenocarcinoma and squamous cell carcinoma groups (*P* = 0.013 and 0.001, respectively). The mean virtual stiffness values among the three groups also reached statistical significance, with *P* values of 0.008 (adenocarcinoma versus non-specific benign) and 0.001 (squamous cell carcinoma versus non-specific benign), respectively. We also compared sADC and virtual stiffness values between malignant and tuberculosis groups. As shown in Supplementary Table, the differences in these two parameters between the two groups were statistically significant. Nevertheless, no significant difference was found among other pathological patterns.

**Table 5:** Comparison of sADC and virtual stiffness values for detailed pathological type

	sADC (×10^− 4^, b = 200,800 s/mm^2^) Mean ± SD (95% C.I.)	virtual stiffness (kPa) Mean ± SD (95% C.I.)
AC (*n* = 26)	6.28 ± 1.70 (5.60–6.97)	9.07 ± 1.33 (8.53–9.61)
SqCC (*n* = 10)	4.90 ± 1.97 (3.49–6.30)	10.21 ± 1.66 (9.03–11.40)
SCC (*n* = 3)	4.31 ± 3.73 (4.95–13.57)	11.27 ± 4.05 (1.20-21.34)
TB (*n* = 4)	7.93 ± 1.02 (6.31–9.54)	7.78 ± 0.79 (6.52–9.05)
Non-specific benign (*n* = 16)	8.60 ± 2.17 (7.45–9.76)	7.14 ± 1.74 (6.21–8.07)
*P* value	< 0.001***	< 0.001***
Post-Hoc Test *P* value	0.013* (AC vs. Non-specific benign) 0.001** (SqCC vs. Non-specific benign)	0.008** (AC vs. Non-specific benign) 0.001** (SqCC vs. Non-specific benign)

Note AC: Adenocarcinoma; SqCC: Squamous cell carcinoma; SCC: Small-cell carcinoma; TB: Tuberculosis

*P-value < 0.05 indicates statistical significance; **P < 0.01; ***P < 0.001

## Discussion

The non-invasive diagnostic vMRE method was first developed and used for liver fibrosis assessments, suggesting a strong correlation between liver tissue elastic properties as revealed with standard MRE, and tissue microstructure as shown with diffusion MR imaging [5]. This correlation was due to the elastic properties of tissues which are linked to the layout of elementary tissue components (i.e., cells, fibers, stroma) to which diffusion MR imaging was exquisitely sensitive [11]. Based on this relationship, vMRE was subsequently applied to hepatocellular carcinoma, liver metastases, non-alcoholic fatty liver disease, pituitary adenoma, and meningiomas, and was considered for potential expansion to the spleen (portal hypertension), breast, prostate (cancer), and brain (neurological disorders) [5]. In this study, vMRE for evaluation of pulmonary neoplasm was proposed to have the potential to indicate stiffness and distinguish property. We present the first study in which malignant neoplasms possessed significantly lower sADC and higher mean virtual stiffness values compared to benign neoplasms. Adenocarcinoma and squamous cell carcinoma were significantly stiffer than non-specific benign neoplasm.

In order to reduce the interference of non-diagnostic percutaneous transthoracic needle biopsy (PTNB) results to the data in this study, we conducted a two-year follow-up of each patient with a pathologically benign diagnosis. Ultimately, two patient were excluded, with a consistent occurrence rate of previous study [12].

A considerable number of studies have used diffusion-weighted imaging (DWI) and IVIM based methods to explore correlations between quantitative parameters, image characteristics, and pulmonary neoplasm. Nevertheless, there have been no deep learning or radiomics studies involving these functional sequences. A meta-analysis on the diagnostic performance of IVIM found that tissue diffusivity (D) value demonstrated the best diagnostic performance and highest post-test probability in the differential diagnosis of lung tumors, followed by the apparent diffusion coefficient (ADC), perfusion fraction (f), and pseudo-diffusivity (D*) values [13]. Other studies involved the differentiation, staging, and proliferation of lung cancer [14], the detection of mediastinal lymph node metastasis [15], the evaluation of lung cancer efficacy, and the prediction of antitumor drug response [16, 17]; all indicated that IVIM-DWI has the potential of comprehensive pulmonary neoplasm evaluation. One previous study reported a significant but moderate correlation of MR elastographic shear modulus with standard ADC and a significant but moderate correlation with the non-Gaussian diffusion index (NGD) [18]. However, the correlation between shear modulus and diffusivity was more prominent with the sADC in which non-Gaussian diffusion effects are incorporated over the ADC. Therefore, sADC is more sensitive to the tissue microstructure compared to ADC [5].

Most studies that have examined lung stiffness were limited to ultrasound elastography. Kuo et al. reported that transthoracic shear-wave ultrasound elastography had the potential of predicting lung malignancy with a suggested cut-off point [19]. Another study revealed that the strain ratio of different pulmonary lesions from low to high included necrosis, atelectasis, consolidation, and tumor [20]. In addition, ultrasound elastography was considered a feasible technique for classifying mediastinal lymph nodes, especially in combination with conventional endobronchial ultrasound imaging [21]. Our current study demonstrated by vMRE that malignant neoplasms were significantly stiffer than benign ones. These results are consistent with prior work on ultrasound elastography.

The reason for this observation may be that increased stiffness of cancer is caused by matrix deposition and remodeling, which can activate signaling pathways that promote proliferation, invasiveness, and metastasis [3, 22]. When normal tissue architecture is disrupted by cancer growth and invasion, microarchitecture is altered [23]. Increased deposition and cross-linking of the extracellular matrix (ECM) lead to matrix stiffening. Increased ECM stiffness and TGF-β signaling activate fibroblasts to become cancer-associated fibroblasts (CAF), initiating a positive feedback loop that further enhances ECM stiffening [24, 25]. Additionally, some collagen fibers are under tension due to cell contraction or local expansion caused by tumor growth [26]. These tensile stresses increase the stiffness of the collagen network, which in turn further activates the focal adhesion contractility of the CAFs in their vicinity, leading to a vicious cycle of matrix deposition and stiffening [27].

Hence, vMRE method may potentially provide a better understanding of microstructural tissue changes in pulmonary neoplasm and enable fast and detailed evaluation of tumor stiffness. Subsequent subtype analyses showed both sADC and virtual stiffness values of adenocarcinoma and squamous cell carcinoma groups were significantly different from non-specific benign group. However, no significant correlation was found in small cell carcinoma and tuberculosis groups. As small cell carcinoma has a faster proliferation rate and reduced extracellular space compared to non-small cell lung cancer, which results in a significant reduction of water diffusion in the tumor, it should theoretically present a higher stiffness. Our negative result regarding small cell carcinoma and tuberculosis may have been due to the small sample size.

There are certain intrinsic limitations to our study. The sample size of the study was not big enough. Therefore, we performed G*power to assess the power of our study. The α vaule was set as 0.05, and the calculated1-β value of sADC and mean virtual stiffness value between malignant and benign groups were 0.996 and 0.842, respectively. Even so, we believe future studies should include larger samples than the small size used here. Moreover, MR lung imaging has limitations. Although the nonrigid registration reduces artifacts caused by motion displacement to a certain extent, it is still difficult to eliminate the influence of respiratory motion. Additionally, objects that change the magnetic field such as calcification may cause certain inaccuracy due to the principle of IVIM-based virtual elastography calculation. Therefore, it follows that the pulmonary neoplasms of the excluded cases are mostly located in the lower lung lobes, where respiratory motion is accentuated.

## Conclusions

In conclusion, our findings provide evidence that malignant neoplasms have significantly lower sADC and higher mean virtual stiffness values than benign ones. There is efficacy in the pathological diagnosis that adenocarcinoma and squamous cell carcinoma are stiffer than non-specific benign neoplasm. To our knowledge, this is the first report of IVIM-based virtual elastography used for pulmonary neoplasm as a promising alternative to invasive biopsy and routine MRE.

### Electronic supplementary material

Below is the link to the electronic supplementary material.

Supplementary Material 1

## Data Availability

The datasets analyzed during the current study are available from the corresponding author on reasonable request.

## Abbreviations

IVIM: Intravoxel incoherent motion
vMRE: Virtual magnetic resonance elastography
sADC: Shifted apparent diffusion coefficient
PTNB: Percutaneous transthoracic needle biopsy
MRE: Magnetic resonance elastography
ROI: Regions of interest
ROC: Receiver operating characteristic
ICC: Intraclass correlation coefficients
FIESTA: T1-weighted fast imaging employing steady-state acquisition
FSE: Fat saturated T2-weighted fast spin echo
DWI: Diffusion-weighted imaging
D: Diffusivity
ADC: Apparent diffusion coefficient
f: Perfusion fraction
D*: Pseudo-diffusivity
NGD: Non-Gaussian diffusion index
ECM: Extracellular matrix
CAFs: Cancer-associated fibroblasts
