# Supplementary material for: Diagnostic efficiency of intravoxel incoherent motion-based virtual magnetic resonance elastography in pulmonary neoplasms
Source: Cancer Imaging. 2024 Jul 6;24:88. doi: 10.1186/s40644-024-00728-1 (PMC11227719; doi:10.1186/s40644-024-00728-1)
Supplement: Supplementary file 1 — Supplementary Material 1 [file 40644_2024_728_MOESM1_ESM.docx]

**Supplementary Table.** sADC and Virtual Stiffness of Malignant Neoplasms and TB

|  | sADC (×10^-4^，b=200,800 s/mm^2^)  Mean±SD (95% C.I.) | virtual stiffness (kPa)  Mean±SD (95% C.I.) |
| --- | --- | --- |
| Malignant(n=39) | 5.78±2.03 (5.12-6.43) | 9.53±1.77 (8.96-10.11) |
| TB (n=4) | 7.93±1.02 (6.31-9.54) | 7.78±0.79 (6.52-9.05) |
| *P* value | 0.024* | 0.027* |

Note: TB: Tuberculosis.

**P* value < 0.05 indicates statistical significance
